# The Book World of Medicine and Science

**Published:** 1900-12-01

**Authors:** 


### The Book World of Medicine and Science.

The Norfolk and Norwich Hospital, 1770 to 1900 By Sir Peter Eade, M.D., F.R.C.P. (Jarrold and Sons. London. 1900. 8vo., pp. 246. Price. 7s. 6d. net).

Under the above title Sir Peter Eade has given us a succinct history of the Institution, and we are thus able to trace clearly the development of the hospital from its primitive stage in 1771 to its evolution in 1900 into one of the finest provincial infirmaries in England. The old building was in the form of the letter H, and it must have been a distinct advance on hospitals of its time for the wards were fairly well lighted and had proper cross ventilation. Its cost was under £14,000. From time to time various additions and alterations were made until 1879, when it was decided to demolish the old building and erect a new one on the old site. This was carried out at a cost of £57,000, and it was opened in 1883. It consists of a series of fine pavilions, having every modern improvement. The author, or compiler, of this volume has, wisely we think, steered clear of the pitfall of attempting to introduce much original matter; and the extracts from the minute book are allowed to tell their own tale. These extracts have been selected with care and judgment. Medical men of the rising generation can hardly realise the difficulties which confronted the staff, medical and lay, of any of the older hospitals in the olden times; and this synopsis of the history of a typical county infirmary should do much to bring these difficulties before the eyes of our younger physicians and surgeons and make them more contented with the age in which they live. What would be thought nowadays of wards dimly lighted with spermaceti oil lamps; or of the water supply being raised from a well by horse-power at one shilling an hour? Some of the extracts from the minute books seem a little quaint. It was ordered that "the Apothecary do provide a suitable nest of drawers to deposit the stones (urinary calculi) extracted in this House, in order to show to strangers, and be referred to occasionally—and none suffered to be taken away." The picturesque was not forgotten, for we find as early as the year 1775 it was ordered that "a blue Livery Coat and Waistcoat, a pair of Leathern Breeches, and a hat with a Yellow Button and Loop, be procured for the Porter."

The example herein set by Sir Peter Eade is one that should be widely followed. Most infirmaries preserve their minute books, but such things may be easily lost or destroyed, and even when in existence and obtainable, few people have patience to wade through fifty volumes of manuscript. This volume is well printed, well indexed, and illustrated. Lists of the physicians and surgeons are given, and among these the well-known names of Alderson, Lubbock, Athill, and Martineau catch the eye.

A Manual of Physiology, with Practical Exercises. By G. N. Stewart, M.A., D.Sc., M.D.Edin., D.P.H.Camb., Professor of Physiology in the Western Reserve University, Cleveland. With 336 illustrations and 5 coloured Plates. Fourth edition. (London: Baillière, Tindall, and Cox. 1900. Price 15s. net.)

The rapid call for a fourth edition of this valuable work shows how well it has been appreciated by those for whose use it is intended. The plan on which the book is arranged is the extremely practical one followed by the author in his teaching in the American university where he is located. Shortly, it is founded on the old maxim that precept and practice should go hand in hand, the arrangement being that each chapter of the systematic course is followed by a series of practical exercises, so that the passage from the detailed discussion of the relations of a phenomenon "to the living fact itself," as is said in the preface, is rendered "easy, natural, and fruitful." Thus in reality the student who adopts this work as his manual reads two books side by side, as in practice the careful student always does—one, namely, on practical and the other on systematic physiology, but with this great advantage, that, instead of his two books being more or less at cross purposes, they are specially arranged chapter by chapter each to illustrate the other. There can, we think, be no doubt that this is a good plan, at any rate where the scheme of teaching adopted in the school accords with that in the book.

Many additions and alterations have been made in this edition, the principal changes being in the chapter on "The Central Nervous System," a subject in which our knowledge advances with such rapidity that physiological works quickly get out of date in this department. The book before us is admirably got up; many of the illustrations are very good indeed, although it must be confessed that some are a little disappointing, and the typographical arrangement by which the importance of the various topics is indicated by the style of type will probably be found useful by the occasional reader. Under the limitation given by the author—namely, that it is to be used for laboratory work under responsible guidance—we think this book will be found of great use to the student.

Consumption and Chronic Diseases. By Emmet Densmore, M.D. London: Swan Sonnenschein & Co. 1900. Pp. 198. 8vo.

Dr. Emmet Densmore, who, though a resident in London, has not his name in the Medical Directory, has used the present craze for the open-air treatment of consumption as a starting point for the discussion of many things, and his own peculiar notions of hygiene in particular.

That there is much of interest and not a little of value in this booklet we freely admit; but the introduction of half-truths, more misleading than falsehoods, rouses in us no small prejudice against the author and his doctrines. Dr. Densmore takes frequent opportunity to insinuate that the orthodox medical man knows little and cares less for hygiene; that he has but one idea in sickness—to give drugs. Dr. Densmore's creed enjoins a scanty use of salt and a diet after the heart of Dr. Lehmann, whose book we not very long ago reviewed in these columns. He is right, of course, in asking for clothing reform and for a greater appreciation of the evils of dust and the virtues of sunlight. But when he declares that by hygienic treatment cancers have been readily removed he becomes misleading and positively mischievous.

